# A case of secondary oxalate nephropathy with nephrotic syndrome

**DOI:** 10.1097/MD.0000000000046524

**Published:** 2025-12-26

**Authors:** Minjie Weng, Chengqin Zha

**Affiliations:** a Department of Cardiology, Beilun People’s Hospital, Ningbo, Zhejiang Province, China; b Department of Nephrology, Beilun People’s Hospital, Ningbo, Zhejiang Province, China.

**Keywords:** nephrotic syndrome, secondary oxalate nephropathy, Traditional Chinese Medicine

## Abstract

**Rationale:**

Nephrotic syndrome, caused by various etiologies and pathophysiological mechanisms, is occasionally complicated by secondary oxalate nephropathy-a rare and often underrecognized condition associated with Chinese herbal medicine use. The co-occurrence of nephrotic syndrome and oxalate nephropathy is extremely uncommon, and delayed diagnosis may lead to severe outcomes.

**Patient concerns:**

A 19-year-old Chinese male presented with persistent edema for over 20 days and significant proteinuria. After taking traditional Chinese medicine for 20 days, his condition worsened rapidly, with new symptoms including infectious fever, erysipelas, acute renal failure, and heart failure.

**Diagnoses:**

The patient was diagnosed with nephrotic syndrome and acute oxalate nephropathy. Renal biopsy confirmed minimal change nephropathy (with possible focal segmental glomerulosclerosis) and acute tubulointerstitial injury with birefringent oxalate crystals in renal tubules.

**Interventions:**

A multidisciplinary treatment approach was adopted, including anti-infective therapy, volume expansion, diuresis, oral vitamin B6, methylprednisolone immunosuppression, and hemodialysis.

**Outcomes:**

After one month of glucocorticoid therapy, hemodialysis, and supportive care, urine output increased and renal function improved. The patient was discharged without requiring further hemodialysis. Outpatient follow-up showed normalized renal function, resolved proteinuria, and normal renal ultrasonography.

**Lessons:**

Although traditional Chinese medicine is widely used in China, its potential to cause secondary oxalate nephropathy is often overlooked. Early renal biopsy is essential for accurate diagnosis and timely intervention in cases of nephrotic syndrome complicated by oxalate nephropathy.

## 1. Introduction

Nephrotic syndrome comprises a constellation of clinical features arising from diverse renal pathologies, characterized primarily by significant proteinuria (≥3.5 g/d), hypoalbuminemia (serum albumin ≤ 30 g/L), hyperlipidemia, and edema. Oxalate nephropathy, an autosomal recessive disorder, stems from defects in glyoxylate and oxalate metabolism. It is defined by primary or secondary causes of hyperoxaluria, clinically manifesting as recurrent urinary calculi, deposition of calcium oxalate in the kidneys, elevated urinary excretion of oxalate, and systemic accumulation of insoluble oxalate deposits. These pathological processes can lead to complications including nephrolithiasis, hematuria, nephrocalcinosis, and acute kidney injury, potentially progressing to chronic renal failure. In oxalate nephropathy, calcium oxalate represents the principal form of tissue deposition, with renal biopsy typically revealing translucent or pale yellow, fan-shaped or cubic angular crystals within renal tubules. These crystals often display partial octahedral morphology and exhibit characteristic light green birefringence under polarized light microscopy.

We present a case of nephrotic syndrome in its early stages. After a 20-day course of traditional Chinese medicine, the patient developed complications including skin infection, severe generalized edema, acute renal failure, and heart failure. Percutaneous renal biopsy revealed minimal change nephropathy with concomitant acute tubular-interstitial injury. Notably, disc-shaped crystals were observed in the lumens of some renal tubules, exhibiting positive birefringence under polarized light, consistent with calcium oxalate deposits, suggesting superimposed oxalate nephropathy. This case highlights the importance of early standardized management of nephrotic syndrome to prevent inappropriate self-treatment. Furthermore, it underscores the necessity of recognizing oxalate nephropathy as a potential complication and demonstrates that early renal biopsy can facilitate timely and accurate diagnosis, thereby guiding appropriate therapeutic intervention.

## 2. Case presentation

On June 5, 2018, a 19-year-old Chinese male was admitted to the hospital presenting with facial and lower extremity edema, right flank and abdominal pain, and fever. The abdominal pain had persisted for more than twenty days. Although initial symptomatic management with gastric protective therapy in the emergency department provided transient relief, his condition progressively deteriorated. He subsequently developed generalized edema, foamy urine, oliguria, and a rapid weight gain of 5 kg within 5 days, along with marked loss of appetite. Repeated urinalysis consistently revealed significant proteinuria (2 + to 3+). Notably, the patient reported a history of self-administering traditional Chinese herbal medicine twice daily for over twenty days, which failed to alleviate his symptoms.

### 2.1. Initial investigations and clinical presentation

1. Laboratory tests on admission:

Blood tests: White blood cell count of 10.6 × 10⁹/L, with neutrophilia (74.5%). Initial serum creatinine was 80 μmol/L.

Imaging studies:

Computed tomography (CT): Revealed extensive bilateral pleural effusions, pulmonary atelectasis, and significant fluid accumulation in the abdominal cavity and retroperitoneal space. Diffuse subcutaneous edema was also noted.

Ultrasonography: Confirmed a large volume of free intra-abdominal fluid and perinephric fluid surrounding the left kidney.

1. Physical examination:

The patient appeared acutely ill and exhibited fever (39.1°C), tachycardia (126 bpm), and hypertension (169/94 mm Hg). Notable physical findings included facial edema, coarse breath sounds with crackles bilaterally on lung auscultation, and severe pitting edema in both lower extremities. A well-demarcated, tender, warm, and erythematous plaque was observed over the right lumbar region and right femoral root. Abdominal examination revealed tenderness and positive shifting dullness, indicative of ascites.

### 2.2. Hospital course and clinical deterioration

1. Progressive complications:

Despite comprehensive management with anti-infective agents, anticoagulation therapy, blood pressure control, and diuretic use, the patient’s clinical condition progressively worsened. He developed anorexia, abdominal distension, chest tightness, and dyspnea on exertion. Renal function declined rapidly over a short period.

1. Follow-up laboratory findings (June 8, 2018):

Severe Renal Impairment: Serum creatinine increased sharply to 513 μmol/L, accompanied by elevated urea (20.9 mmol/L) and uric acid levels (529 μmol/L).

Additional Laboratory Abnormalities: Markedly elevated C-reactive protein (164.4 mg/L), increased D-dimer (5573 μg/L), and dyslipidemia were observed.

1. Further diagnostic evaluations:

Renal ultrasonography: Revealed sonographic features consistent with acute nephropathy, including bilateral renal enlargement, loss of corticomedullary differentiation, and increased cortical echogenicity.

Vascular imaging: Demonstrated elevated resistive indices in the renal arteries and diffuse soft tissue edema in the lower extremities.

The patient subsequently developed anuria, met diagnostic criteria for acute kidney injury (AKI), and exhibited signs of decompensated heart failure, ultimately requiring initiation of hemodialysis.

### 2.3. Treatment and clinical course

The treatment approach was multifaceted:

1. Discontinuation: The use of traditional Chinese herbal medicine was immediately discontinued.
2. Immunosuppressive therapy: High-dose intravenous methylprednisolone was initiated at 80 mg daily, with subsequent tapering to 40 mg daily based on clinical response.
3. Anticoagulation: Low molecular weight heparin and batroxobin were administered to manage hypercoagulability and elevated D-dimer levels.
4. Renal support: Hemodialysis was promptly initiated to address severe AKI and fluid overload.
5. Symptomatic management: Included diuretics, blood pressure control, and correction of electrolyte imbalances.

Response to treatment: Over the subsequent 20 days, the patient exhibited a progressive increase in urine output and marked improvement in renal function. Hemodialysis was successfully weaned and discontinued, and the patient was transitioned to oral prednisone at a dose of 60 mg daily. He was discharged in significantly improved condition.

Definitive diagnosis: Renal Biopsy

**Figure 1.**
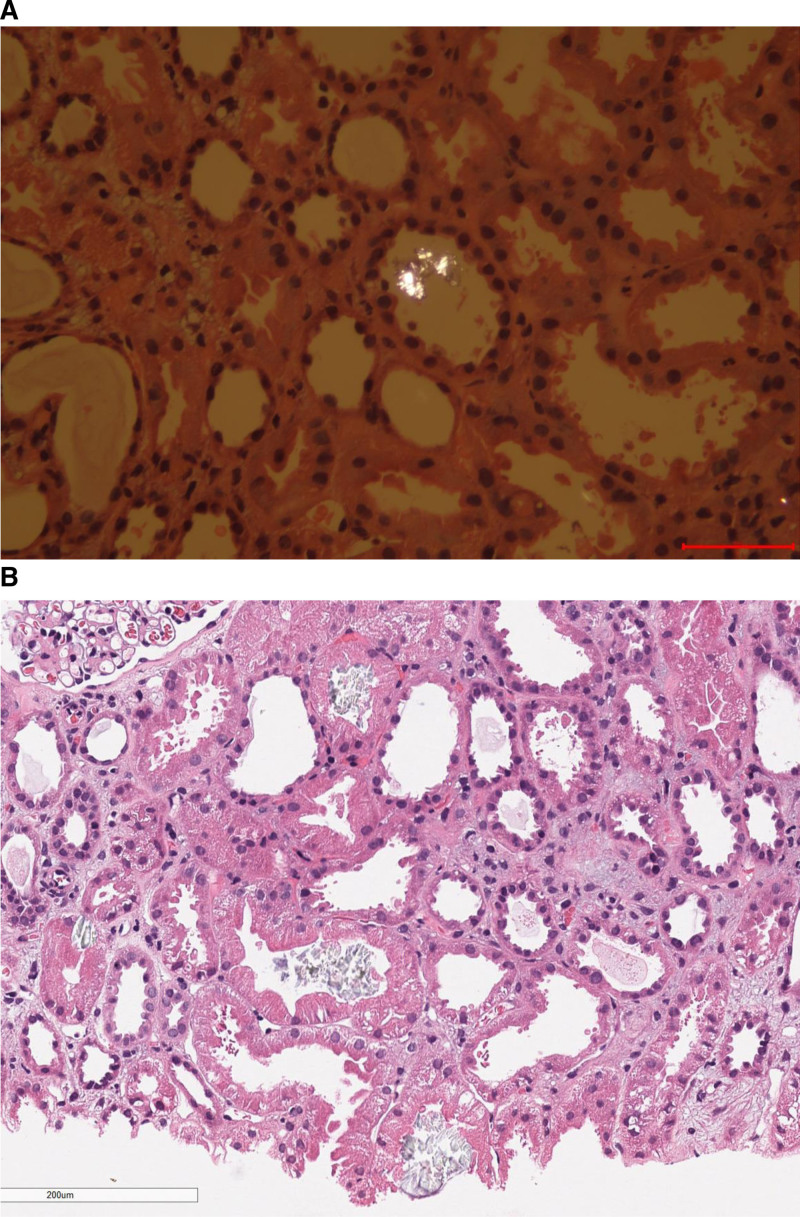
Oxalate crystals in renal-biopsy tissue. Renal-biopsy tissue samples with polarized staining (A) and hematoxylin and eosin staining (B) show oxalate crystals.

A renal biopsy performed on June 22, 2018, established the definitive diagnosis as follows:

1. Minimal change nephropathy (with focal segmental glomerulosclerosis not excluded).
2. Acute tubulointerstitial injury with oxalate nephropathy, characterized by the presence of birefringent crystals within the renal tubules(Fig. 1).

### 2.4. Outcome and follow-up

During an outpatient follow-up visit on July 28, 2018, the patient’s plasma albumin levels, renal function, and urinary protein excretion had all returned to normal ranges. Renal ultrasonography revealed no structural abnormalities. All medications were successfully discontinued approximately 6 months after the follow-up, with sustained remission observed thereafter.

## 3. Discussion

The patient, a male adolescent, presented with an acute onset of clinical features including marked proteinuria, hypoalbuminemia, severe edema, and hyperlipidemia, leading to a confirmed diagnosis of nephrotic syndrome. Prior to admission, he had developed oliguria, skin infection, and heart failure. Shortly after admission, his condition rapidly deteriorated to anuria and AKI. Renal biopsy revealed minimal change disease with concomitant acute tubulointerstitial injury, showing round, disc-shaped crystals within the lumina of renal tubules that exhibited positive birefringence under polarized light microscopy-findings characteristic of oxalate deposition. Integrating the clinical history, laboratory data, and histopathological evidence, the final diagnosis was oxalate nephropathy secondary to Chinese herbal medicine use, occurring in the context of nephrotic syndrome and AKI.

The patient’s grandfather, a practitioner of traditional Chinese medicine, administered herbal remedies for over 20 days following disease onset. No other medications or dietary supplements were reported. Notably, many of these herbal formulations are derived from plants high in oxalic acid, which likely led to excessive oxalate accumulation in renal tissues, resulting in acute tubulointerstitial injury and subsequent oxalate nephropathy. The patient experienced recurrent back pain, and urinary ultrasonography revealed acute nephritic changes, with no evidence of renal vein thrombosis or urinary calculi. The response to glucocorticoid therapy was delayed, attributable to persistent tubulointerstitial damage caused by oxalate-induced tubular obstruction.

Following comprehensive management, including hemodialysis, blood pressure control, correction of acid-base imbalance, fibrinolytic anticoagulation therapy, lipid-lowering treatment, vitamin B6 supplementation, and full-dose glucocorticoid immunosuppression, the patient’s urine output and renal function gradually improved. He was subsequently discharged with marked clinical improvement. Outpatient follow-up more than 10 days after discharge showed complete normalization of renal function and urinary parameters. Glucocorticoid therapy was discontinued approximately 6 months after initiation.

Urinary oxalate excretion in healthy individuals is <0.5 mmol/(1.73 m²·d). In patients with oxalate nephropathy, however, urinary oxalate levels are markedly elevated, frequently exceeding 1 mmol/(1.73 m²·d). When renal function is preserved, plasma oxalate concentrations typically remain within the normal range. Once the glomerular filtration rate falls below 60 mL/(min·1.73 m²), however, plasma oxalate levels begin to rise.^[1–5]^ Accumulated oxalate tends to deposit in tissues with high calcium content, such as the kidneys, heart, blood vessels, and bones, resulting in progressive multiorgan damage. Oxalate generated in the systemic circulation is filtered by the kidneys and concentrated approximately 10 to 50 folds within the renal tubules. This elevated intratubular oxalate concentration exerts direct cytotoxic effects on renal tubular epithelial cells, leading to cellular injury.^[6]^

Oxalate nephropathy is classified into primary hyperoxaluria (PH) and secondary oxalate nephropathy. PH is an autosomal recessive disorder resulting from inborn errors of glyoxylate and oxalate metabolism. Genetic testing is essential for the definitive diagnosis of PH.^[3–5]^ Patients with PH typically present at a young age with recurrent urinary calculi, renal calcium oxalate deposition, progressive decline in renal function, and eventual progression to end-stage renal disease. A detailed family history often reveals a genetic predisposition, and these patients generally have a poor prognosis. Notably, disease recurrence may occur even after kidney transplantation. Combined liver-kidney transplantation represents a potential curative approach for severe cases^[7,8]^; however, its application is limited by factors such as economic burden and donor availability. Currently, gene therapy shows promise for patients with PH, but further clinical evidence is required to establish its efficacy and feasibility in routine practice.

Secondary oxalate nephropathy presents with nonspecific clinical manifestations, including fatigue, anorexia, gastrointestinal disturbances, edema, and reduced urine output or anuria. Laboratory findings typically show elevated serum creatinine levels, increased urinary oxalate excretion, and normal renal size and contour on ultrasonography.^[3–5]^ In cases complicated by thrombosis or inflammatory exudation, bilateral renal enlargement may be observed, although overt urinary tract symptoms are generally absent. A definitive diagnosis is established by renal biopsy, which reveals calcium oxalate crystal deposition within the renal tubules. Patients often have a history of consuming oxalate-rich foods or excessive vitamin C intake.

The use of traditional Chinese medicine is widespread across various regions, particularly in Asia, and has gained increasing recognition in global healthcare systems due to its long-standing clinical practices and holistic therapeutic approaches. Despite the extensive use of herbal remedies, there are few published reports of their adverse renal effects. Sharma et al identified herbal medications as the most common cause of drug-induced AKI at a center in Northeast India.^[9]^ Kumar et al described a series of 3 cases of tubulointerstitial nephritis associated with different herbal medications in central India.^[10]^ Ashok Bhat et al reported 4 cases of herbal medicine-induced renal damage, further highlighting the potential nephrotoxicity of these agents.^[11]^

Renal involvement associated with herbal medications manifests in various forms, including AKI, renal tubular functional defects, electrolyte disturbances, hypertension, chronic kidney disease, papillary necrosis, nephrolithiasis, and urothelial malignancies.^[12,13]^

In this case, the development of oxalate nephropathy was strongly associated with prolonged use of traditional Chinese medicine. Contributing factors included the patient’s poor appetite, concomitant nephrotic syndrome, markedly reduced oral intake of food and fluids, and inadequate effective blood volume. These conditions collectively promoted systemic oxalate deposition and precipitated the onset of renal injury.

The prognosis of oxalate nephropathy is closely associated with the timely identification and accurate diagnosis of its underlying causes. Clinicians should maintain a high index of suspicion for this condition and conduct a thorough investigation of potential etiologies during clinical evaluation. Key steps include:

- Comprehensive assessment of dietary habits, family history, and past medical conditions;
- Evaluation for gastrointestinal or pancreaticobiliary disorders;
- Early renal biopsy in suspected cases without contraindications, to confirm the diagnosis and prevent misdiagnosis or diagnostic oversight.

Once the underlying cause is identified, prompt intervention can be initiated to eliminate contributing factors, slow the progression of renal failure, and improve patient outcomes.

## 4. Conclusions

Traditional Chinese Medicine is widely used in China; however, due to limited clinical awareness, its potential to induce secondary oxalate nephropathy is frequently overlooked or misdiagnosed. Although rare, this condition may contribute to otherwise unexplained cases of renal failure. Early renal biopsy is crucial for establishing an accurate diagnosis and facilitating timely therapeutic intervention in patients with secondary oxalate nephropathy associated with nephrotic syndrome. Early discontinuation of the offending agent can help in preventing progression. Short course of corticosteroids can be helpful in reversing the renal damage as seen in our case.

## Acknowledgments

We thank Kingmed Diagnostics for providing pathological analysis support.

## Author contributions

**Writing – original draft:** Chengqin Zha.

**Writing – review & editing:** Minjie Weng.
